# Management of coexisting patent foramen ovale and pulmonary arteriovenous malformation: a case report of sequential closure

**DOI:** 10.1093/ehjcr/ytaf045

**Published:** 2025-01-29

**Authors:** Hiroto Yagasaki, Takeki Suzuki, Keitaro Watanabe, Ryota Watanabe, Toshiyuki Noda

**Affiliations:** Department of Cardiology, Gifu Prefectural General Medical Center, 4-6-1, Noisshiki, Gifu 500-8717, Japan; Department of Medicine, Indiana University School of Medicine, 340 West 10th Street, Fairbanks Hall, Suite 6200, Indianapolis, IN 46202-3082, USA; Department of Medicine, Indiana University School of Medicine, 340 West 10th Street, Fairbanks Hall, Suite 6200, Indianapolis, IN 46202-3082, USA; Department of Cardiology, Gifu Prefectural General Medical Center, 4-6-1, Noisshiki, Gifu 500-8717, Japan; Department of Cardiology, Gifu Prefectural General Medical Center, 4-6-1, Noisshiki, Gifu 500-8717, Japan; Department of Cardiology, Gifu Prefectural General Medical Center, 4-6-1, Noisshiki, Gifu 500-8717, Japan

**Keywords:** Patent foramen ovale, Pulmonary arteriovenous malformation, Cryptogenic stroke, Platypnoea–orthodeoxia syndrome, Paradoxical embolism, Percutaneous closure, Case report

## Abstract

**Background:**

Concurrent patent foramen ovale (PFO) and pulmonary arteriovenous malformation (PAVM) are rare but can cause paradoxical embolism and platypnoea–orthodeoxia syndrome (POS).

**Case summary:**

A 72-year-old female with embolic stroke history developed positional dyspnoea. Evaluation revealed right-to-left shunting through PFO and PAVM in the right middle lobe. Orthodeoxia was confirmed by 6% SpO_2_ decrease upon position change. A staged approach was adopted: PFO closure with Amplatzer™ Occluder, followed by PAVM embolization 1 month later. Symptoms improved significantly post-procedure. No residual shunting or symptoms have been observed during the 2-year follow-up.

**Discussion:**

This case emphasizes thorough evaluation in patients with cryptogenic stroke and POS, especially when symptoms persist. It demonstrates the effectiveness of staged treatment for concurrent PFO and PAVM, highlighting the importance of individualized strategies and long-term follow-up.

**Learning points**

- Treatment sequence for concurrent PFO and PAVM should be individualized based on predominant clinical presentation.
- Persistent right-to-left shunting after PFO closure warrants careful evaluation of pulmonary veins for concurrent PAVM.

## Introduction

Cryptogenic stroke accounts for 30%–40% of ischaemic strokes, with paradoxical embolism through right-to-left shunts being a key mechanism.^1^ Patent foramen ovale (PFO) and pulmonary arteriovenous malformations (PAVMs) are two such shunt pathologies.^1–3^ Both can cause platypnoea–orthodeoxia syndrome (POS), characterized by positional dyspnoea and hypoxaemia.^4^ The coexistence of PFO and PAVM is uncommon, presenting diagnostic and treatment challenges.^5–11^ Few reported cases exist, and optimal management strategies remain unclear. We present a case of concurrent PFO and PAVM in a patient with cryptogenic stroke and POS, demonstrating evaluation and closure techniques for complex right-to-left shunts.

## Summary figure

**Figure:**
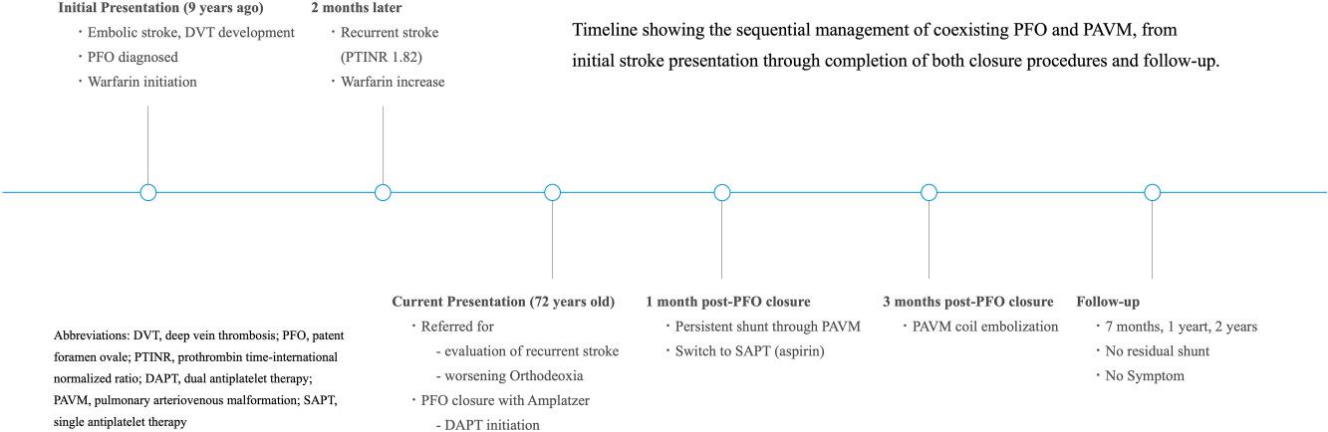


## Case presentation

A 72-year-old Asian woman with a history of embolic stroke and hypertension was referred for evaluation of recurrent stroke aetiology and symptoms of shortness of breath upon standing or changing positions. Nine years earlier, the patient presented to the emergency room due to transient rotatory vertigo and nausea. Brain MRI revealed high-intensity areas in the right insular cortex extending to the frontal lobe, corona radiata, and left insular cortex. Lower extremity venous ultrasound showed deep vein thrombosis. Transoesophageal echocardiography (TEE) revealed a PFO with continuous left-to-right shunting. The patient was diagnosed with paradoxical cerebral embolism and started on warfarin (maintenance dose 3.0 mg/day). Two months later, despite anticoagulation (PT-INR 1.83; below therapeutic range of 2.0–3.0), a follow-up brain MRI showed new infarctions, indicating recurrent cerebral infarction. Given the presence of DVT requiring higher-intensity anticoagulation and the patient’s stable PT-INR control, warfarin was continued and increased to 4.0 mg/day rather than switching to alternative anticoagulation strategies. The patient reported experiencing positional shortness of breath for several years before the recurrent strokes, attributing it to aging. This symptom had gradually worsened over time.

**Figure 1:**
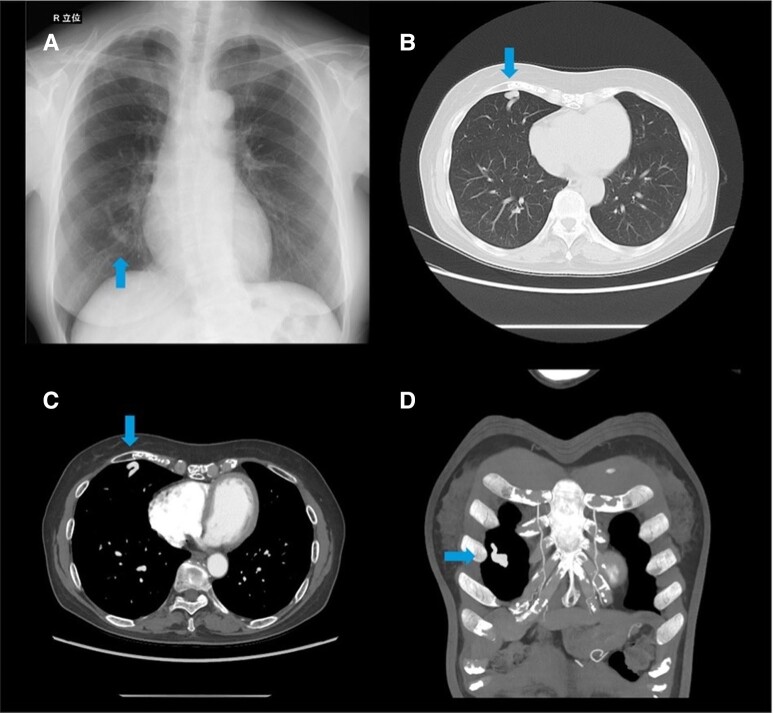
Pulmonary arteriovenous malformation. (*A*) Chest X-ray: increased vascular markings in the right lower lung (arrow). (*B–D*) Chest CT: pulmonary arteriovenous malformation (blue arrow) (*B* non-contrast; *C*, *D* contrast enhanced).

On current presentation, the patient’s vital signs were as follows: blood pressure 142/90 mmHg, heart rate 77 b.p.m., and oxygen saturation 98% while sitting and 92% upon standing. Physical examination revealed normal cardiopulmonary findings without murmurs. Neurological examination was unremarkable. Laboratory findings showed PT-INR 2.92 (therapeutic range adjusted for age: 1.6–2.6), NTproBNP 460 pg/dL (normal range: <125 pg/dL), and D-dimer 0.50 μg/mL (normal range: <1.0 μg/mL). Electrocardiogram revealed normal sinus rhythm at 60/min. Chest X-ray showed a cardiothoracic ratio of 43.4% without congestion or effusion. An enlarged pulmonary vessel was noted in the right lower lung field (*Figure 1A*).

**Figure 2:**
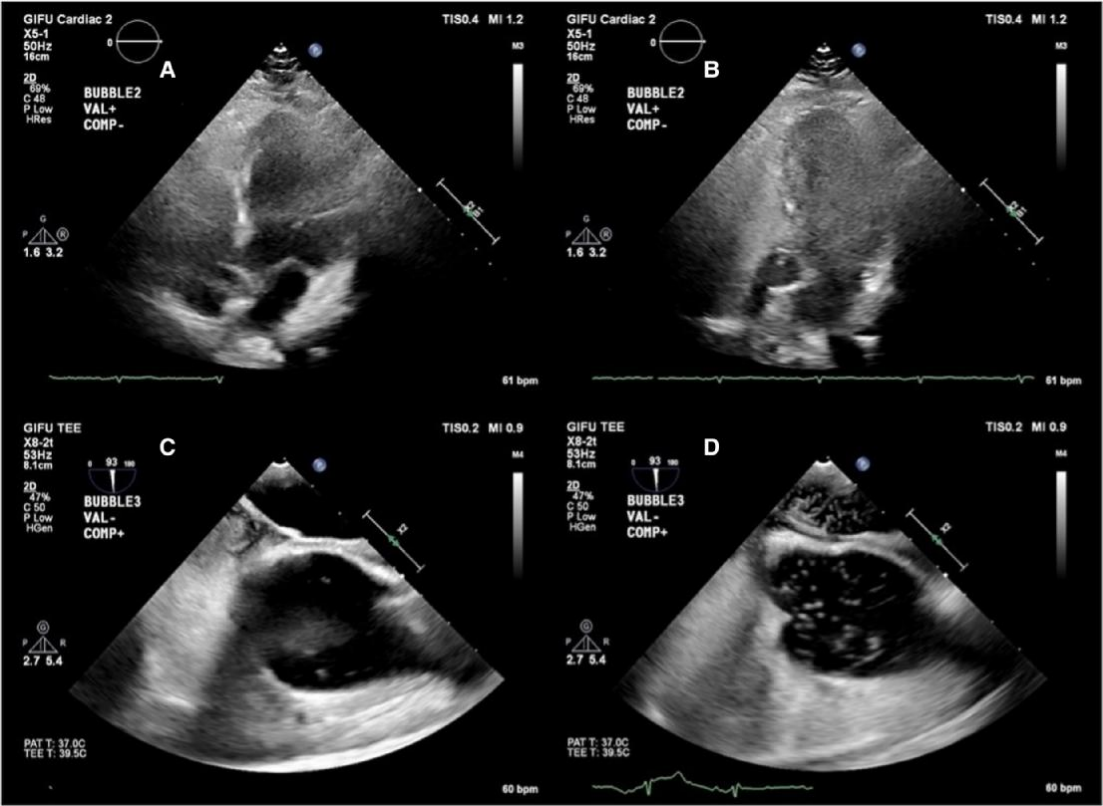
Bubble study before PFO closure. (*A*, *B*) Transthoracic echocardiography immediately after (*A*) and 3 cycles after (*B*) bubble injection. (*C*, *D*) Transoesophageal echocardiography immediately after (*C*) and 3 cycles after (*D*) bubble injection.

Chest CT with contrast revealed a PAVM in the right middle lobe, with feeding and draining vessels ∼4 mm in diameter (*Figure 1B–D*). Brain CT and MRI showed changes consistent with the patient’s previous stroke, without new abnormalities. Transthoracic echocardiography (TTE) and TEE confirmed the presence of PFO but showed no other significant abnormalities. Bubble studies under Valsalva manoeuvre with TTE and TEE demonstrated significant right-to-left shunting. Transthoracic echocardiography with Valsalva manoeuvre showed grade 3 bubbles within three cardiac cycles, increasing to grade 4 after four cycles (*Figure 2A* and *B*; Supplementary material online, *Video S1*).

Transoesophageal echocardiography revealed grade 3 bubbles within three cardiac cycles at rest and during abdominal compression release (*Figure 2C* and *D*; Supplementary material online, *Video S2*). Orthodeoxia was confirmed by a 6% decrease in SpO_2_ decrease from standing to sitting.

**Figure 3:**
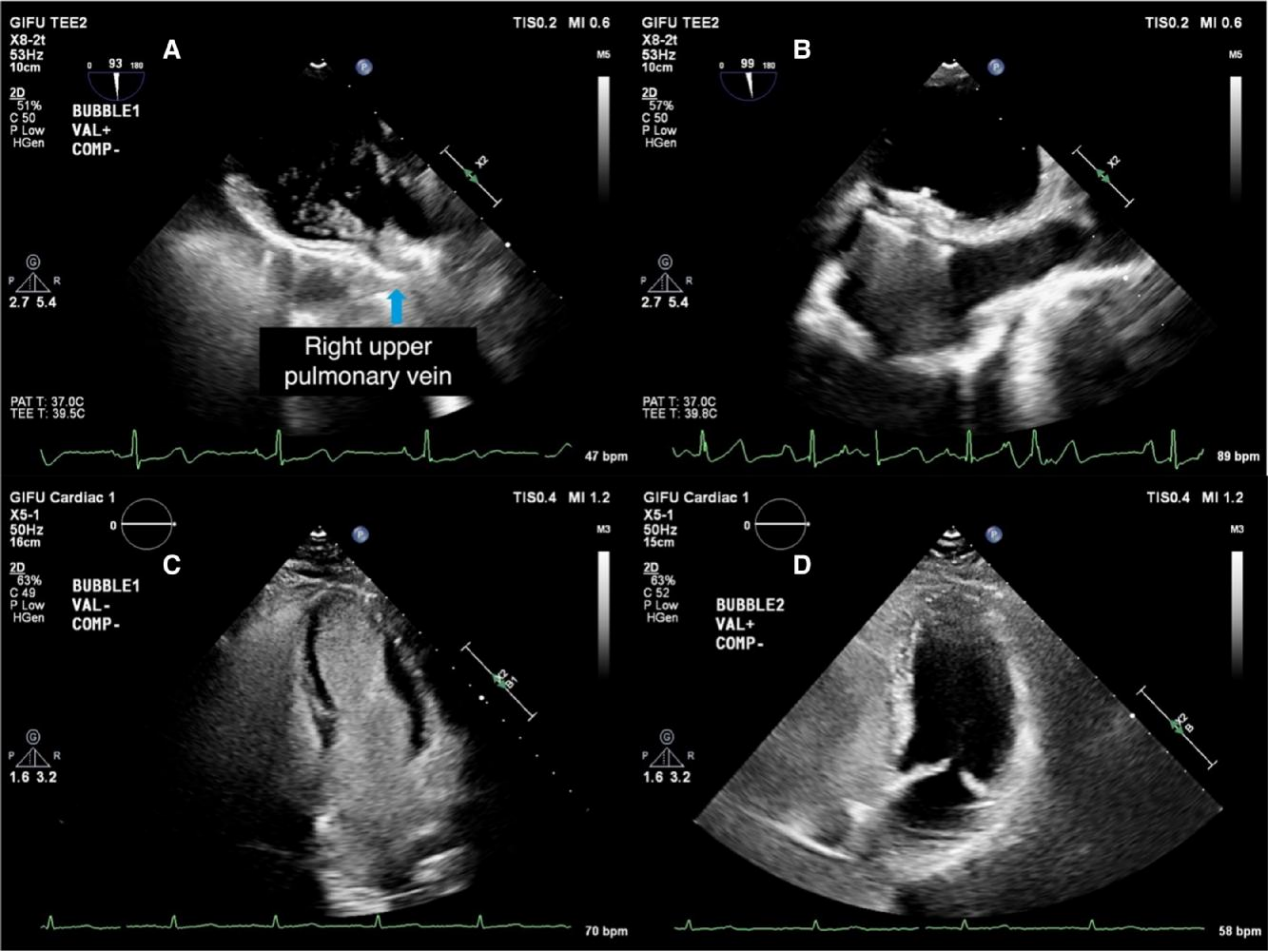
Intraoperative transoesophageal echocardiography and follow-up transthoracic echocardiography during patent foramen ovale and pulmonary arteriovenous malformation closure. (*A*) Intraoperative transoesophageal echocardiography: grade 3 bubble flow from the right upper pulmonary vein to the left atrium. (*B*) Intraoperative transoesophageal echocardiography: post-patent foramen ovale closure with occlusion device. (*C*) Transthoracic echocardiography post-patent foramen ovale closure, pre-pulmonary arteriovenous malformation closure: grade 4 left heart opacification >4 cycles after right heart. (*D*) Transthoracic echocardiography 4 months post-pulmonary arteriovenous malformation closure: no left heart bubble flow.

Percutaneous PFO closure was performed under general anaesthesia with TEE. Intraoperative bubble study revealed significant right-to-left shunting through both PFO and right lower pulmonary vein (*Figure 3A*; Supplementary material online, *Video S3*). A 25 mm Amplatzer™ PFO Occluder (Abbott Medical Plymouth, MN, USA) was deployed (*Figure 3B*).

**Figure 4:**
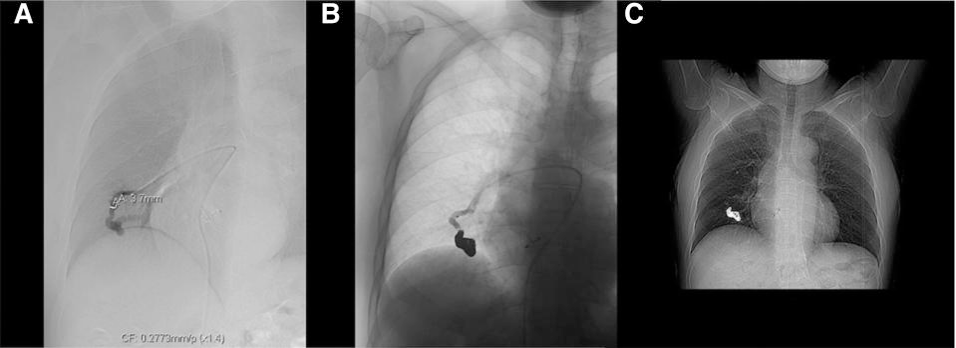
Pulmonary arteriovenous malformation closure procedure. (*A*) Selective angiography of pulmonary arteriovenous malformation before closure. (*B*) Selective angiography of pulmonary arteriovenous malformation after closure, showing no contrast passage. (*C*) Chest X-ray after pulmonary arteriovenous malformation closure.

One month post-PFO closure, bubble studies confirmed persistent shunting through the right lower pulmonary vein, suggesting PAVM (*Figure 3C*; Supplementary material online, *Video S4*). At this time, antiplatelet therapy was switched from dual antiplatelet therapy (aspirin 100 mg/day and clopidogrel 75 mg/day) to single antiplatelet therapy (aspirin 100 mg/day). The patient reported improved positional dyspnoea and SpO_2_. Percutaneous coil embolization of the PAVM was performed at another hospital 3 months after post-PFO closure (*Figure 4A–C*). Single antiplatelet therapy was continued through and after the PAVM closure. Follow-up evaluations at 7 months post-PFO closure and at 1 and 2 years post-procedures showed no significant residual shunting (*Figure 3D*; Supplementary material online, *Video S5*). The patient remained symptom free. Follow-up CT scans showed no recanalization.

## Discussion

This presents the 11th reported case of concurrent PFO and PAVM treated by percutaneous procedures (*Table 1*, 5–11). Patent foramen ovale is present in 25%–30% of the general population and accounts for 5% of all ischaemic strokes, rising to 10% in patients under 55 years.^12,13^ In contrast, PAVM is a rare vascular anomaly with an estimated prevalence of 0.025%.^2,3^ It carries a stroke risk of 9%–18%, and modelling studies suggest that by age 65, at least 25% of untreated individuals with PAVM will have experienced a clinical stroke.^3^ This in prevalence and stroke association rates indicates that PAVM likely carries a higher individual risk for paradoxical embolism compared to PFO.

**Table 1:** Literature review of cases with coexisting patent foramen ovale and pulmonary arteriovenous malformation

No.	Year	Author	Age	Sex	Presenting symptoms	Medical history	Timing of PAVM detection	Simultaneous procedure	Notes
1	2003	Schussler *et al*.^5^	27	Female	Transient ischaemic attack (aphasia and confusion)	Epilepsy, migraine	After PFO closure	–	PAVM detected by TTE bubble study after PFO closure, showing persistent LA bubble entry.
2	2005	Peters *et al*.^6^	41	Female	Transient ischaemic attack	Left hemispherical stroke	After PFO closure	–	PAVM detected by TEE bubble study showing left PV bubble entry and pulmonary angiography. Chest X-ray and CT were not diagnostic.
3	2008	Gaspardone *et al*.^7^	40	Female	Persistent left-sided migraine with visual and sensory aura, transient ischaemic attack, seizures	–	Before PFO closure	+	PFO detected by TEE, PAVM detected by chest X-ray. PFO and PAVF closed simultaneously.
4	2016	Kijima *et al*.^8^	72	Female	Two episodes of stroke	–	After PFO closure	–	PAVM detected by TTE bubble study after PFO closure, showing persistent LA bubble entry.
5	2018	Shah *et al*.^9^	50	Male	Stroke	–	After PFO closure	No data	—
6	2018	Shah *et al*.^9^	55	Female	Stroke	–	After PFO closure	No data	—
7	2018	Shah *et al*.^9^	33	Female	Stroke	–	After PFO closure	No data	—
8	2018	Shah *et al*.^9^	42	Male	Stroke	–	After PFO closure	No data	—
9	2020	Liu and Yang^10^	48	Female	Right hemiparesis, dysphasia	–	After PFO closure	–	PAVF detected by contrast-enhanced CT after post-PFO closure stroke.
10	2021	Park *et al*.^11^	51	Female	Focal cortical infarction in the left occipital area (right hemifield visual defect)	Epilepsy, migraine	Before PFO closure	+	TEE showed LA bubble entry from non-PFO source, suggesting another shunt. PAVF confirmed by contrast-enhanced CT. Closed simultaneously with PFO.
11	2024	Yagasaki *et al*.	72	Female	Two episodes of stroke (dizziness), short of breath upon position change	Hypertension	Before PFO closure	–	Coexisting POS was identified.

+, present; −, absent

While 94% of PAVMs in Caucasians were reported to be associated with hereditary haemorrhagic telangiectasia (HHT),^3^ a study on Asian populations reported a lower rate of 38%, suggesting genetic influence.^14^ In our case, only one criterion for HHT was met, indicating a low probability of coexistence.

Imaging modalities, particularly echocardiography with bubble studies, are crucial in investigating ischaemic stroke aetiology.^1^ Bubble studies are effective in detecting less apparent shunts, with the timing of contrast appearance differentiating intracardiac from extracardiac shunts.^1^ Our case highlights the importance of careful pulmonary vein assessment during TEE bubble studies, enabling accurate diagnosis of concurrent PAVM and PFO.

A study of 560 patients who underwent PFO closure found that 0.7% were later diagnosed with PAVM requiring closure, an incidence higher than the general population prevalence of 0.025%.^9,12^ This observation underscores the importance of comprehensive evaluation and follow-up in PFO patients, particularly when symptoms or positive bubble studies persist post-closure.

The decision for PFO closure in this case provides insights into individualized treatment approaches. The indication for PFO closure is typically based on the Risk of Paradoxical Embolism (RoPE) score.^1^ Generally, patients aged 18–60 years with a RoPE score of 7 or higher are considered candidates.^1^ In this case, the patient was 63 at onset with a RoPE score of 6, and 72 with a score of 5 when treatment was considered. However, the patient experienced shortness of breath during positional changes, consistent with POS. Over 80% of POS cases are caused by intracardiac shunts such as atrial septal defect or PFO.^4^ Guidelines suggest that PFO closure may be considered for patients with POS.^1^ The improvement in positional dyspnoea and SpO_2_ decrease after PFO closure validates this approach.

Indications for PAVM closure include a feeding artery diameter of 3 mm or greater, or the presence of symptoms such as paradoxical embolism.^15^ In this case, both criteria were met. Repeated CT scans and bubble studies showed no worsening up to 1.5 years post-treatment, and the improvement in shortness of breath has been maintained.

The optimal approach for treating concurrent PFO and PAVM remains unclear. In our case, PFO closure was prioritized due to longstanding POS symptoms. However, PAVM closure might be prioritized in some cases, given the higher risk of cerebral embolism and other serious complications. This case demonstrates the effectiveness of a staged approach in managing concurrent PFO and PAVM, emphasizing comprehensive evaluation and individualized treatment in complex cases with persistent symptoms.

## Supplementary Material

ytaf045_Supplementary_Data

## Data Availability

The data underlying this article are available in the article.

## Lead author biography

Dr Hiroto Yagasaki is a cardiologist at Gifu Prefectural General Medical Center, Japan, and a visiting researcher at Indiana University School of Medicine, USA. He specializes in echocardiography and cardiac magnetic resonance imaging, focusing on structural heart disease interventions. At his institution in Japan, he leads procedures including transcatheter aortic valve implantation, edge-to-edge valve repair, left atrial appendage closure, and patent foramen ovale closure. He holds board certification in internal medicine, cardiology, echocardiography, and other cardiovascular subspecialties in Japan.

## Notes

**Consent:** The patient reviewed and provided written informed consent for publication in compliance with COPE guidelines.

**Funding:** None declared.
